# Virome Profiling of Chickens with Hepatomegaly Rupture Syndrome Reveals Coinfection of Multiple Viruses

**DOI:** 10.3390/v15061249

**Published:** 2023-05-26

**Authors:** Guoshuai Wang, Yaqi He, Xiaomin Yan, Yue Sun, Le Yi, Changchun Tu, Biao He

**Affiliations:** 1 Changchun Veterinary Research Institute, Chinese Academy of Agricultural Sciences, Changchun 130122, China; wgs1142394873@163.com (G.W.); xiaomin_yan0716@163.com (X.Y.); sy3126999@163.com (Y.S.); loyee1988@foxmail.com (L.Y.); changchun_tu@hotmail.com (C.T.); 2 Beijing Centre Biology Co., Ltd., Beijing 102600, China; zhangjiali102@163.com; 3 Jiangsu Co-Innovation Center for Prevention and Control of Important Animal Infectious Diseases and Zoonosis, Yangzhou 225009, China

**Keywords:** virome, chicken, hepatomegaly rupture syndrome, avian encephalomyelitis virus, experimental animal infection

## Abstract

Liver diseases seriously challenge the health of chickens raised on scaled farms and cause tremendous economic losses to farm owners. The causative agents for liver diseases are still elusive, even though various pathogens, such as the hepatitis E virus, have been reported. In the winter of 2021, a liver disease was observed on a chicken farm in Dalian, China, which increased chicken mortality by up to 18%. We conducted panvirome profiling of the livers, spleens, kidneys, and recta of 20 diseased chickens. The viromic results revealed coinfection of multiple viruses, including pathogenic ones, in these organs. The viruses were highly identical to those detected in other provinces, and the vaccine and field strains of avian encephalomyelitis virus (AEV) and chicken infectious anemia virus (CIAV) cocirculated on the farm. In particular, the liver showed higher abundance of AEV and multiple fowl adenoviruses than other organs. Furthermore, the liver also contracted avian leukemia virus and CIAV. Experimental animals with infected liver samples developed minor to medium lesions of the liver and showed a virus abundance profile for AEV across internal organs similar to that in the original samples. These results suggest that coinfection with multiple pathogenic viruses influences the occurrence and development of infectious liver disease. The results also highlight that strong farm management standards with strict biosafety measures are needed to minimize the risk of pathogenic virus introduction to the farm.

## 1. Introduction

Chickens constitute an essential part of global animal husbandry. However, liver diseases have caused huge economic losses to the intensive chicken farming industry [1,2]. Indeed, such diseases not only increase broiler mortality by 1–4% but also reduce laying hen egg production by 10–40% in some cases [2,3,4]. Among these liver diseases, the infectious ones caused by pathogens are the most concerning, such as hepatitis–splenomegaly syndrome (HSS), big liver and spleen disease (BLSD), and hepatic rupture hemorrhage syndrome (HRHS) [4,5,6,7,8,9]. HRHS causes bleeding in the abdomen, liver hemorrhage, and splenomegaly, with increased mortality and decreased laying rates in chickens [9]. HSS results in regressive ovaries, red fluid in the abdomen, and enlarged liver and spleen [5]. BLSD induces splenomegaly and hepatomegaly and results in decreased egg production and increased mortality in chickens [4,10].

Viruses from different families can cause viral hepatitis in poultry. Hepatitis E virus (HEV) is associated with HSS, BLSD, and HRHS in chickens, resulting in liver swelling, softening, hemorrhaging, etc. [11,12,13,14]. Fowl adenoviruses (FAdVs) are divided into five species, some of which are responsible for inclusion body hepatitis and hepatitis-hydropericardium syndrome in chickens, which are characterized by swollen, friable, discolored, and even necrotic livers [15,16]. Visceral Marek’s disease (MD) caused by MD virus and lymphoid leukemia caused by avian leukemia virus (ALV) lead to liver enlargement with tumors in chickens [17,18]. Chicken infectious anemia virus (CIAV) can result in anemia in chickens with thymus atrophy, bone marrow yellowing, and liver necrosis [19,20]. In addition, although not noted in chickens, some viruses can cause severe viral hepatitis in other poultry, such as duck hepatitis caused by duck astrovirus and duck hepatitis A/B viruses and turkey viral hepatitis caused by turkey hepatitis virus [21].

However, these viruses cannot explain all causes of the intricate disease because, in some cases, chickens with hepatitis are negative for these pathogens while chickens with the pathogens usually remain healthy [5,22]. Therefore, the cause of infectious chicken hepatitis needs to be further clarified. Currently, approaches used in the clinical diagnosis of this disease mainly focus on specific pathogens, resulting in the complete virus spectrum of chickens with this disease being rarely investigated [23,24,25]. Unbiased viral metagenomic sequencing provides an indispensable method for profiling the virome and has been shown to be very effective in the precision surveillance of animal diseases [26]. For the first time, we conducted virome profiling of chickens with infectious liver disease in this study. Follow-up PCR/RT-PCR validation, phylogenetic analysis, and experimental animal infection provided additional insights into the cause of this disease.

## 2. Materials and Methods

### 2.1. Sample Collection

In the winter of 2021, there was a sudden increased mortality on a large chicken farm in Dalian City. The in-house veterinarian examined the dead chickens and found that they featured severe liver damage but with symptoms not typical for HSS, BLSD, and HRHS. The veterinarian collected the livers, spleens, kidneys, and recta of 20 dead chickens for virome analysis, but the liver and spleen samples from one chicken were missing. Samples were cryotransported to our laboratory, where they were stored at −80 °C.

### 2.2. Sample Pretreatment and Viral Metagenomic Sequencing

To obtain a complete spectrum of viruses infecting the chickens, the samples were subjected to metavirome analysis using a combination of metatranscriptome (MTT) and multiple displacement amplification (MDA) techniques. To inspect any possibilities of cross-contamination, five specific pathogen-free (SPF) chickens were used as controls. The protocol for sample pretreatment and viral metagenomic sequencing was published previously [27]. Briefly, every five tissues (~0.2 g per each) of the same organ were mixed, which resulted in 16 pools for follow-up processing. These samples were sequentially subjected to homogenization, clarification, filtration, and digestion [27]. The resultants of each pool were allocated into two groups for RNA and DNA extraction. For MTT, total RNA was subjected to rRNA depletion and then to RNAseq library preparation. For MDA, total DNA was magnified before sequencing. All libraries were sequenced on an Illumina NovaSeq 6000 sequencer at Novogene Co., Ltd. (Beijing, China).

### 2.3. Virome Annotation

Raw data were first quality controlled using fastp version 0.19.7 and then mapped against the host genome assembly (GenBank accession number: GCA_016699485.1) using bowtie2 version 2.4.1 to remove host genome sequences. A fast metagenomic classification of reads was performed using Kraken2 version 2.0.9-beta to assign bacteria, archaea, and fungi. The unassigned reads were mixed according to the library type and de novo assembled using SPAdes genome assembler version 3.14.1 with the mode of meta. Contigs of ≥500 nt were queried against the Eukaryotic Viral Reference Database (EVRD) version 2.0 [28] using blastn and diamond blastx with an e-value cutoff of 1 × 10^−10^. Then, the unassigned reads were mapped against virus-like contigs (VLCs) to check the chimeric assembly and calculate the vertical and horizontal coverage using SAMtools version 1.10.

### 2.4. PCR/RT-PCR Detection

Based on the virome results, we designed primer pairs to detect FAdV-B, FAdV-D, FAdV-E, avian encephalomyelitis virus (AEV), ALV, and CIAV. The primer sequences are available upon request. We cut ~0.2 g of each sample and extracted the total RNA and DNA using an RNeasy Mini kit (Qiagen, Dusseldorf, Germany) and a DNeasy Blood and Tissue kit (Qiagen, Dusseldorf, Germany), respectively. RNA was reverse transcribed into cDNA using a PrimeScript II 1st strand cDNA Synthesis Kit (TaKaRa, Dalian, China). PCR was carried out using 2 × MasterMix (Tiangen, Beijing, China) with double distilled water as a negative control. The PCR program was initiated by predenaturation at 94 °C for 3 min, followed by 35 cycles of denaturation at 94 °C for 30 s, annealing at 53 °C (or adjusted according to different primer pairs) for 30 s, extension at 72 °C for 40 s, and ended with a final extension at 72 °C for 7 min. The products were checked by electrophoresis using 1.0% agarose gel with the expected one being directly sequenced on an ABI 3730xl DNA analyzer (Comatebio, Changchun, China).

### 2.5. Histological Section Preparation

The liver with a severe lesion was cut into ~8 cm^3^ pieces and fixed in 4% paraformaldehyde. The fixed samples were dehydrated using ethanol, followed by immersion and embedding in paraffin. Embedded tissues were cut into 5 μm thick sections and stained using hematoxylin and eosin. Each section was observed using an Eclipse Ci-L microscope (Nikon, Tokyo, Japan).

### 2.6. Virus Isolation Using Chicken Embryos

To reduce the bias introduced by individual samples, we cut ~0.2 g tissues from each liver, pooled them together, and then subjected them to homogenization using sterile PBS supplemented with 100,000 U/mL penicillin and 10,000 g/mL streptomycin (Invitrogen, Carlsbad, CA, USA). The samples were then centrifuged at 10,000× *g* for 5 min, and the supernatants were filtered through 0.45 μm pore size membranes (Millipore, Boston, MA, USA). Then, 200 μL of filtrates were inoculated into 10-day-old SPF embryonated chicken eggs via the allantoic cavity using a standard egg inoculation procedure. This inoculation was performed in triplicate. The inoculated eggs were incubated at 37 °C for three days with daily inspection. Allantoic fluids (AFs) were harvested and subjected to three rounds of inoculation. AFs at each passage were inspected for AEV, FAdV, and CIAV using the PCR/RT-PCR methods described above.

### 2.7. Experimental Animal Infection

A total of 20 SPF 20-week-old chickens were purchased from Harbin Veterinary Research Institute. They were randomly divided into four groups, with each having five animals. Chickens within one group were treated as blank controls and sacrificed to check the initial health status of their internal organs. Their livers, spleens, kidneys, lungs, brains, and recta were collected and subjected to virome analyses and histological section preparation as described above. Filtrates used to inoculate eggs were orally (1.5 mL for each chicken) or intravenously (500 μL for each chicken) inoculated into two groups, which were designated as OI and II. They, along with the negative control (chickens inoculated with sterile water), were individually raised in cages contained in a 25 m^2^ clean room to ensure no physical contact. These chickens were fed twice a day, and their food intake, defecation, behavior, etc. were inspected. All chickens were sacrificed at 10 d.p.i. and subjected to examination of the organ lesions. Their livers, spleens, kidneys, brains, lungs, and recta were collected for virome analysis. The livers from chickens with a severe lesion in the OI group and the negative control were used for histological section preparation.

### 2.8. Phylogenetic Analyses

The genome structure of contigs was first predicted using open reading frame (ORF) Finder and then compared to their genetic neighbors. These contigs were queried against GenBank, and their reference sequences were downloaded for phylogenetic analyses. The multiple sequence alignment was generated using mafft L-INS-I, and the ambiguous parts were trimmed using TrimAL. The best evolutionary model was predicted using ModelFinder with the Akaike information criterion. The maximum likelihood phylogeny was reconstructed using MEGA version 7.0 (https://megasoftware.net, accessed on 15 May 2019) and examined using the bootstrap test with 1000 replicates. The pairwise similarity between sequences was calculated using MegAlign (DNASTAR, Madison, WI, USA).

## 3. Results

### 3.1. Overview of the Disease Outbreak

**Figure 1:**
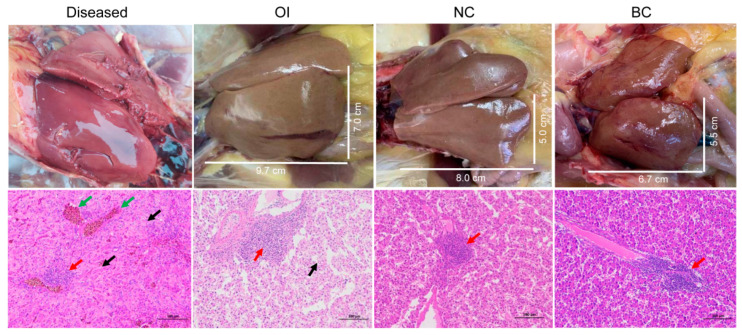
Gross pathology (upper panel) and histological section (lower panel) of chicken livers in the diseased, orally infected (OI), negative control (NC), and blank control (BC) groups. The liver section of diseased chickens showed intensive hepatocyte necrosis, karyolysis, cytoplasmic disintegration, and homogeneous red staining (eosinophil infiltration, arrowed in black). Lymphocytes accumulate around blood vessels (arrowed in red) and veins and hepatic sinuses were extensively congested (arrowed in green). The livers from OI chickens also showed severe degeneration, swelling, and deformation of hepatocytes and vacuolation of the cytoplasm (arrowed in black). The portal area was infiltrated by inflammatory cells (arrowed in red). However, the livers in the NC and BC groups appeared normal with very mild lymphocyte infiltration (arrowed in red).

Since October 2021, an infectious liver disease has been recurring on a chicken farm in Dalian City, China. The farm has raised ~260,000 Arbor Acres Plus broiler breeders and had not experienced any overt diseases in the last year before that. These chickens have been vaccinated against a series of viral pathogens, such as avian infectious laryngotracheitis virus, avian influenza virus, Newcastle disease virus, AEV, and CIAV. The disease struck chickens older than 12 weeks, killing 3–5% of those aged 12–19 weeks old, but mortality increased to 15–18% in those older than 25 weeks. The necropsy of those dead animals showed abnormal livers in >90% of the cases. The main features were hepatomegaly, hepatic steatosis, liver fragility, glandular gastric papilla hemorrhage, and thickened mesentery (Figure 1). These syndromes did not resemble HSS, BLSD, or HRHS; hence, we tentatively named the disease hepatomegaly rupture syndrome (HMRS). A routine examination using microscopy and PCR revealed positivity of Escherichia coli in the liver and spleen, Salmonella in the gut, Clostridium in the duodenum, and Vibrio campylobacter in the liver. The histological section showed massive necrotic hepatocytes, karyolysis, cytoplasmic disintegration, infiltration of eosinophils and lymphocytes around vessels, and congestion of liver sinuses (Figure 1), indicating severe liver damage.

### 3.2. Virome Profile of Different Organs

**Figure 2:**
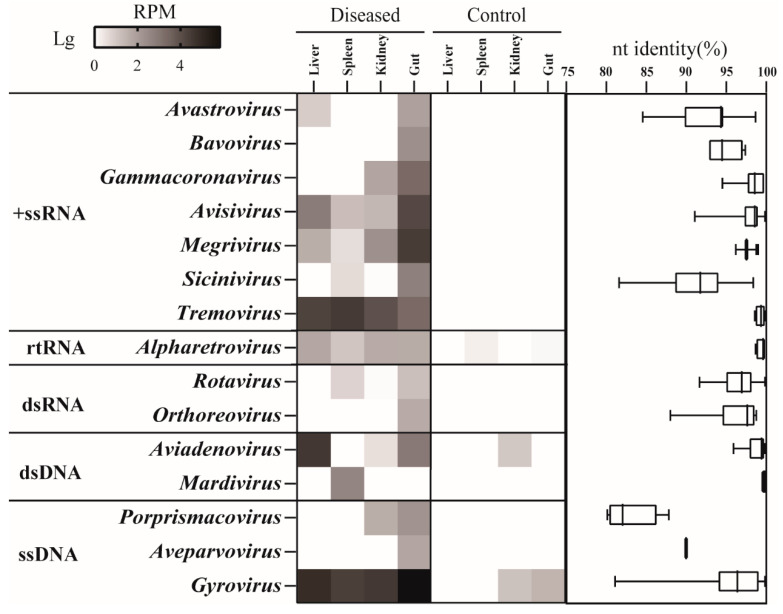
Virome overview of chickens in the diseased and control groups. Left panel, heatmap of viral reads cataloged into 15 genera (RPM, reads per million total reads; +ssRNA, single-stranded positive RNA; rtRNA, reverse-transcribing RNA; dsRNA, double stranded RNA; dsDNA, double-stranded DNA; ssDNA, single-stranded DNA). Right panel, nucleotide identity range of the assembled contigs in the given viral genera with reference sequences in GenBank. Each box plot illustrates the estimated median (centerline), upper and lower quartiles (box limits), and outliers (points) of similarity.

The liver, spleen, kidney, and rectum of 20 dead chickens were subjected to virome profiling using a combination of MTT and MDA techniques (Table S1). The two methods generated 129.2 and 136.1 gigabases of data, respectively. After removal of host genomic sequences and contamination by bacteria, archaea, and fungi, the remaining reads were de novo assembled into 10,505 contigs. The virome annotation revealed 368 DNA and 148 RNA VLCs. These VLCs were 500–20,253 nt long and annotated to 15 genera within 10 families (Figure 2). Ten genera were RNA viruses, such as *Avastrovirus*, *Gammacoronavirus*, *Tremovirus*, and *Rotavirus*. The remaining genera belonged to double-stranded *Aviadenovirus* and *Mardivirus* and single-stranded *Porpsrismacovirus*, *Aveparvovirus*, and *Gyrovirus*. We used SPF chickens as negative controls, which revealed very limited viruses, indicating no cross-contamination between samples. A comparison of the virus genus richness and abundance in these organs showed the recta encompassed almost all genera except *Mardivirus*, with *Avisivirus* and *Megrivirus* having the highest abundance. Of note was that *Tremovirus*, *Aviadenovirus*, and *Gyrovirus* showed higher abundance in livers than in other samples. In addition, *Tremovirus* was also very abundant in spleens. All these VLCs were closely related to known viruses with >80% nucleotide (nt) identity. In particular, contigs of *Gammacoronavirus*, *Avisivirus*, *Megrivirus*, *Tremovirus*, *Alpharetrovirus*, *Aviadenovirus*, and *Mardivirus* showed extremely high identities (>95%) to their genetic neighbors. The virome results did not show any HEV signals.

### 3.3. Phylogenetic Diversity

**Figure 3:**
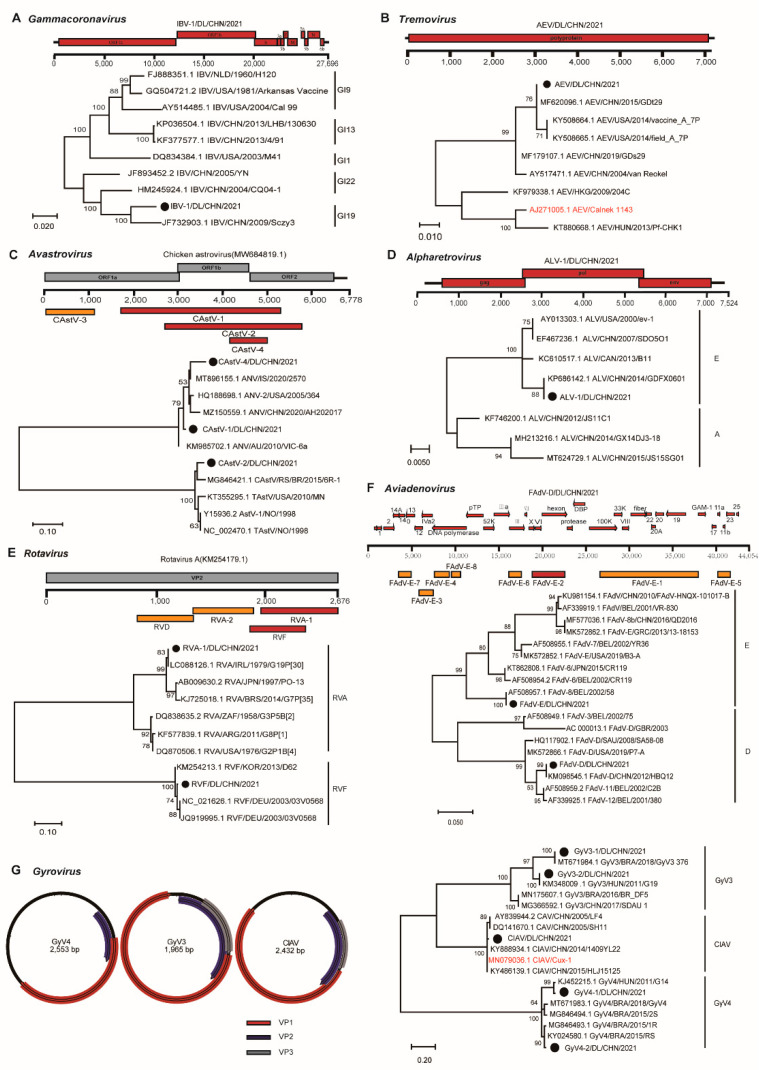
Distribution of contigs annotated into the genera *Gammacoronavirus* (**A**), *Tremovirus* (**B**), *Avastrovirus* (**C**), *Alpharetrovirus* (**D**), *Rotavirus* (**E**), *Aviadenovirus* (**F**), and *Gyrovirus* (**G**) against reference sequences and their (filled circles) phylogenetic relationships with other representatives. These phylogenies are based on the nucleotide sequences of ORF1b (**A**), polyprotein (**B**), contig CAstV-4 and its counterparts (**C**), pol (**D**), contig RVF and its counterparts (**E**), contig FAdV-E-2 and its counterparts (**F**), and VP1 (**G**). The contigs of AEV, ALV, FAdV-D, GyV, and CIAV cover complete genomes and are directly used to illustrate their genomic structures. Contigs annotated into the same virus are indicated in the same color. Vaccine strains in the phylogenies of AEV and CIAV are highlighted in red.

The virome profile revealed several viruses that are known to be pathogenic to chickens. Thus, we conducted phylogenetic analyses of viruses from seven genera based on their contig length and pathogenicity. We obtained a complete genome of infectious bronchitis virus (IBV) by de novo assembly and gap-filling PCR, which belonged to the genus *Gammacoronavirus*. IBV results in an acute, highly contagious respiratory disease in chickens and severely affects the egg production of laying hens [29]. The contig (IBV-1/DL/CHN/2021) was 27,652 nt long and showed 97.7% nt identity with IBV Sczy3 (Figure 3A), which was isolated from the kidney of a diseased chicken in Sichuan Province [30]. Avian encephalomyelitis virus (AEV) belongs to the genus *Tremovirus* within the family *Picornaviridae*. The contig of AEV (AEV/DL/CHN/2021) was 7129 nt long, covering the entire polyprotein. The phylogenetic analysis based on the ORF revealed its extremely high identity (99.5%) with GDt29 (Figure 3B), an AEV detected from Guangdong Province, but it only shared 82% nt identity with the vaccine strain used on the farm (Figure 3B). Among the four avastrovirus contigs, three overlapped at the region covering partial ORF1b and ORF2 (Figure 3C). These overlaps were very divergent (57–80% nt identities) from each other and fell into two clades, but all of them were very similar to their genetic neighbors with 85–93% nt identities (Figure 3C). Although we found diverse retroviruses, we only focused on ALV as it leads to tumors in chickens [31]. The contig of ALV (ALV-1/DL/CHN/2021) covered all ORFs and fell into clade E with as high as 99.7% nt identity with an ALV isolated from Guangdong Province (Figure 3D). Rotaviruses (RVs) are a group of segmented double-stranded RNA viruses with many members that cause diarrhea in animals [32]. We found coinfection of group A RV (RVA), RVD, and RVF on the farm. They were also closely related to known viruses with 90–99% nt identities (Figure 3E).

Among these DNA viruses, FAdVs are divided into five species or 12 serogroups, with many members causing varying diseases in chickens [33]. The virome showed circulation of FAdV B, D, and E on the farm. In particular, we obtained the complete genome of FAdV D (FAdV-D/DL/CHN/2021), which was 44,056 bp long. Phylogenetic analysis based on the hexon gene showed that these FAdVs were very similar to known viruses with 99.5–99.9% nt identities (Figure 3F). We discovered diverse circular DNA viruses within the genus *Gyrovirus* of the family *Anelloviridae*. The genus *Gyrovirus* has 11 members, with CIAV being an important pathogen for chicks [34,35]. These gyrovirus (GyV) contigs were grouped into three clades with 80.0–99.0% nt identities with their genetic neighbors (Figure 3G). The CIAV contig was 98.5% identical to a virus isolated from Heilongjiang Province and 98.3% identical to the vaccine strain used on the farm (Figure 3G).

### 3.4. PCR/RT-PCR Detections

The virome revealed infection of several pathogenic viruses in the liver, including AEV, FAdV B/D/E, ALV, and CIAV. We conducted PCR/RT-PCR detection to validate these viruses as well as HEV, which was absent in the virome (Table S2). The results showed that AEV was positive in all liver and spleen samples except two livers but only positive in 60% of kidneys and 65% of rectum samples. The three FAdVs showed similar positivity in livers (~33%) and in recta (~30%), which was higher than in kidneys, in which only FAdV B was detected in 20.0% of the samples. In the spleen, FAdV B and E also showed high prevalence (~34%), but FAdV D was detected in only one sample. ALV and CIAV were positive in all samples except in a rectum and two kidneys where ALV and CIAV were negative, respectively. RT-PCR detection further validated the negativity of HEV in these samples.

### 3.5. Experimental Animal Infection

We attempted to isolate the viruses by inoculating 10-day-old chicken embryos with liver samples. After three blind passages, PCR/RT-PCR detection did not obtain any positive results for AEV, FAdV B/D/E, ALV, and CIAV, suggesting failure in virus isolation using chicken embryos. We further infected SPF chickens using liver samples via two means, namely, oral infection (OI) and intravenous infection (II), to test whether the syndrome could be replicated. Before infection, we checked that these SPF chickens were extremely healthy with no abnormalities in their organs or in histological sections of the liver and spleen. Some infected animals in the two groups showed varying depressions at 1 d.p.i. but recovered quickly. All animals had normal food intake and weights during the 10-day observation. However, compared to the negative controls, animals in the two infected groups had minor to medium degrees of perianal feather loss and liquid feces between 5 and 10 d.p.i. The necropsy showed that three OI and two II chickens had livers 10–20% larger than those of the blank and negative controls (Figure 1). In addition, the livers in the two groups turned yellowish brown with scattered hemorrhagic spots on the capsular surface and became loose. However, other organs, including the spleen, remained normal, as did the organs in the negative controls. Generally, the complete check showed that chickens in the OI group were more characteristic, so we examined the histological sections of a liver with the most severe abnormality in the OI group. The results showed extensive hepatocyte degeneration, swelling, and deformation (Figure 1). The cell cytoplasm in the liver began to vacuolate, and many inflammatory cells infiltrated the portal area. However, these sections of the negative control showed relative normality except for slight lymphocyte infiltration (Figure 1). These sections from the blank control showed a small number of focal infiltrates for inflammatory cells that were around the blood vessels (Figure 1).

**Figure 4:**
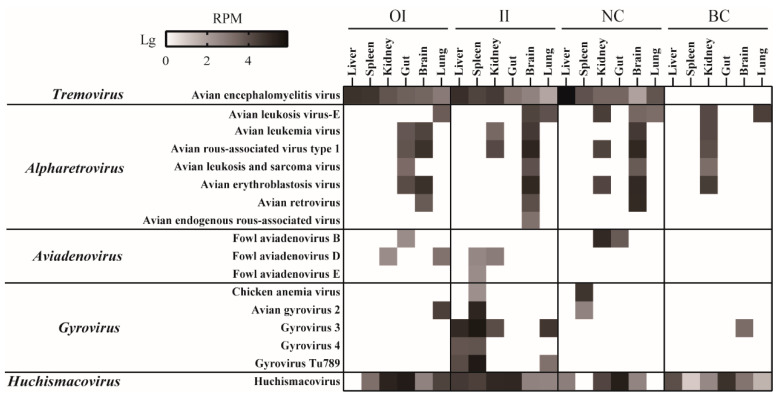
Virome overview of chickens in orally infected (OI), intravenously infected (II), negative control (NC), and blank control (BC) groups.

The livers, spleens, kidneys, brains, lungs, and recta of the chickens in the two infected groups and the negative and blank controls were subjected to virome profiling. The blank controls did not reveal any other viruses, except for retroviruses and non-CIAV small circular DNA viruses (Figure 4). However, AEV, CIAV, and FAdV B/D/E were all or partially detected in the two infected groups and the NC group. The detection of these viruses in the NC group should be attributed to the fecal–oral transmission during the co-house feeding. Surprisingly, AEV showed similar abundance profiles in the livers, spleens, kidneys, brains, lungs, and recta from the three groups as in the original clinical samples, i.e., more abundant in the liver/spleen than in other organs (Figure 4). The virome did not find any CIAV signals in the organs of the OI group but did find them in the spleens from the II and negative control groups (Figure 4). FAdVs were not detected in any liver samples but were detected in the spleens from the chickens in the II group (Figure 4).

## 4. Discussion

Here, we profiled the whole virome for chickens that died from HMRS and infected chickens for experimentation. The findings revealed that the chickens were infected with a variety of viruses, including pathogenic viruses, such as IBV, FAdV, AEV, CIAV, and rotaviruses. As expected, the guts had the richest diversity of virus species compared to other solid organs (Figure 2). These chickens featured liver diseases; hence, we focused on the unique characteristics of the liver virome. The presence of tremovirus and FAdVs was greater in the liver and/or spleen than in other organs (Figure 2), which caught our interest. FAdVs can cause liver disease [36,37,38,39]. In addition, the virome also showed that the livers were coinfected with CIAV and ALV, which are also related to liver damage [9]. We detected these viruses using PCR/RT-PCR in all samples, which was highly consistent with the viromic results, i.e., viruses with greater abundance in the liver were considerably more prevalent in the liver than in the other organs. Notably, we did not find HEV infection in these animals, even though the virus is widely recognized as the causative agent of infectious liver diseases [25]. Although this study cannot determine the genuine cause of the disease, it is reasonable to infer that AEV, FAdV, CIAV, and ALV jointly participated in the development of HMRS in the chickens.

AEV is an important pathogen in chickens and is spread by fecal–oral and vertical routes [40,41]. Chicks are very sensitive to the virus and develop neurological diseases when infected, including tremors, ataxia, and difficulties walking due to progressive weakness or paralysis of the legs [42]. Field strains of AEV mainly replicate in the intestine and pancreas, leading to a drop in egg production in laying hens [43]. However, studies have also shown that the virus was detected in a variety of organs in experimentally infected quails with high viral loads in the proventriculus, spleen, and bursa [44]. By virome profiling and RT-PCR detection of the original samples, we demonstrated that AEV also targets the liver/spleen of chickens. In addition, the virus successfully infected SPF chickens by oral and intravenous infection and circulated within all organs with the same replication dynamics as in the original samples, i.e., higher abundance in the liver/spleen than in other organs (Figure 4). Interestingly, the chickens in the negative control group were also infected by the viruses, and the viruses showed higher abundances in the liver, which could be attributed to the fecal–oral transmission of the virus. However, the virome examination of the experimental chickens did not reveal FAdV, CIAV, ALV, and other virus infections in the liver, although FAdVs were found in the spleen, kidney, and gut of certain individuals (Figure 4). We further inspected the histological sections of the liver and found that, compared to the normal livers in the blank control, livers in the two infected groups showed minor lesions (Figure 1). Although it is premature to conclude that such lesions were caused by AEV infection, these data provide additional insights into the occurrence and development of HMRS as well as the replication and pathogenicity of AEV in chickens, i.e., AEV can infect the liver and likely causes lesions to the organ. Indeed, it is not surprising that AEV could be liver pathogenic given that some members within the family *Picornaviridae* are known to be important causative agents of viral hepatitis in poultry, such as duck hepatitis A virus and turkey hepatitis virus [21]. However, other experiments, especially those that fulfill Koch’s postulates, are needed to confirm that AEV is a hepatitis-causing agent. The first step should be to isolate the virus by tissue culture, but we failed to do so by embryonic inoculation, which could be due to biotoxin interference and/or the inadaptability of these viruses to embryos. The liver pathogenicity should then be tested to see if it can be replicated by infection with the isolate.

Notably, we found cocirculation of the AEV and CIAV field and vaccine strains in the samples, although these chickens were vaccinated against the two viruses before the disease outbreak. This can be largely asserted as vaccination failure. In large-scale farming, vaccination is the main measure in the control and prevention of infectious diseases. However, there are many factors interrupting the effect of vaccination, such as vaccine quality and procedure. Vaccine effectiveness should be assessed in a timely manner to ensure that it works. In addition, phylogenetic analyses revealed that some viruses, e.g., AEV, rotavirus, and FAdV, were extremely similar to those circulating in other provinces, indicating cross-regional transmission of these pathogens. Thus, high standards of farm management with strict biosafety measures are needed to minimize the risk of pathogen virus introductions into the farm.

In summary, this study demonstrates the coinfections of pathogenic viruses in chicken livers from chickens that died from HMRS. Furthermore, these chickens were also positive for multiple bacteria. Accordingly, multiple infections with viruses and bacteria were very likely the main cause of death for these chickens as bacterial diseases such as colibacillosis, fowl typhoid, and erysipelas are also related to liver lesions in poultry. In addition to pathogens, many other noninfectious factors also seriously challenge the liver health of these intensively farmed chickens. For example, mycotoxins produced by fungi and excessive use of drugs in diets could result in irreversible liver damage [45]. Long-term consumption of diets with an unbalanced protein-to-energy ratio leads to fatty liver syndrome [46]. Moreover, genetic selection of high productivity also endows some strains with the propensity to develop liver diseases [45]. Accordingly, integrated strategies such as balanced feeding, sound management, stringent biosafety, and efficient vaccination must be considered for liver protection.

## Data Availability

The complete genomes of the three viruses were deposited in GenBank under accession numbers OQ749505-OQ749511. The viral metagenomic sequencing raw data are available in the NCBI Sequence Read Archive (SRA) under accession number PRJNA923241.

## Supplementary Materials

The following supporting information can be downloaded at https://www.mdpi.com/article/10.3390/v15061249/s1, Table S1: Read numbers of different viruses in these samples; Table S2: Results of PCR/RT-PCR detection in these clinical samples.
